# A database of handwriting samples for applications in forensic statistics

**DOI:** 10.1016/j.dib.2019.105059

**Published:** 2019-12-31

**Authors:** Amy Crawford, Anyesha Ray, Alicia Carriquiry

**Affiliations:** Iowa State University, USA

**Keywords:** Handwriting, Forensics, Statistics, Pattern evidence, Image analysis

## Abstract

Handwriting samples were collected from 90 adults for the purpose of developing statistical approaches to the evaluation of handwriting as forensic evidence. Each participant completed three data collection sessions, each at least three weeks apart. At each session, a survey was completed and three writing prompts were each transcribed three times. In total, the repository includes 2430 handwriting sample images as well as demographic and session specific information for all 90 participants. The writing samples were scanned and instructional header text was cropped out to obtain the raw writing data as image files. Survey data are provided in table format. Reliable methods for data management were incorporated through systematic document generation, QR code text embedding, and the development of an application to facilitate data entry and automated file naming and handling. The data presented in this article were collected by researchers at the Center for Statistics and Applications in Forensic Evidence (CSAFE) at Iowa State University.

**Table:** Specifications Table

Subject	Decision Sciences: Statistics, Probability, and Uncertainty
Specific subject area	Statistical analysis of forensic pattern evidence. Statistical foundations for the analysis and interpretation of forensic evidence.
Type of data	Table Image
How data were acquired	Individuals over the age of 18 who are able to read and write in English provided handwriting samples and filled out a short survey on three different data collection occasions, or sessions. These sessions were separated by at least three weeks in time. Participants were encouraged to enrol and complete their sessions in groups.
Data format	Raw (images are raw handwriting scans, x pixels chopped off the top to remove instructional information)
Parameters for data collection	Participants received packets including a blue BIC pen, instruction sheet, survey page, and 9 pages of paper with small headers indicating the prompt to be written in the space below. The first of three sessions for each participant was facilitated by a researcher, either in-person or via conferencing software. Packet distribution was done via mail or in person depending on participant location. Once completed, the session packets were returned to researchers and scanned at 300dpi using an Epson DS-6500 document scanner.
Description of data collection	At each session, participants were asked to complete a survey and transcribe three repetitions of each of three writing prompts, resulting in 9 writing samples per session. At the end of the study, each participant contributed 27 writing samples and three surveys.
Data source location	United States, mostly in Iowa/Midwest.
Data accessibility	Repository name: CSAFE Handwriting Database. Hosted at Iowa State University's DataShare Repository. Direct URL to data: https://doi.org/10.25380/iastate.10062203

**Table:** 

**Value of the Data** • These data facilitate the development of probabilistic methods for evaluation of handwritten evidence. • This repository may benefit researchers doing similar forensic statistics focused work, or forensic document examiners, interested in examining repeated writing over time or investigating the relation between demographic or situational information and the writing. Outside of forensic evaluation, there may be benefit for researchers developing or testing optical character recognition methods, or for anyone looking to use images of this nature for broader image analysis techniques. • Forensic handwriting analysis is used to evaluate the source of ransom notes, forgeries, and other such pieces of handwritten evidence. The dataset serves as a sample to build statistical approaches to handwriting analysis. These data, and the methods based upon them, have potential implications in the judicial process, to ensure a transparent, accessible, repeatable, and more objective consideration of handwritten evidence.

## 1 Data

**Fig. 1:**
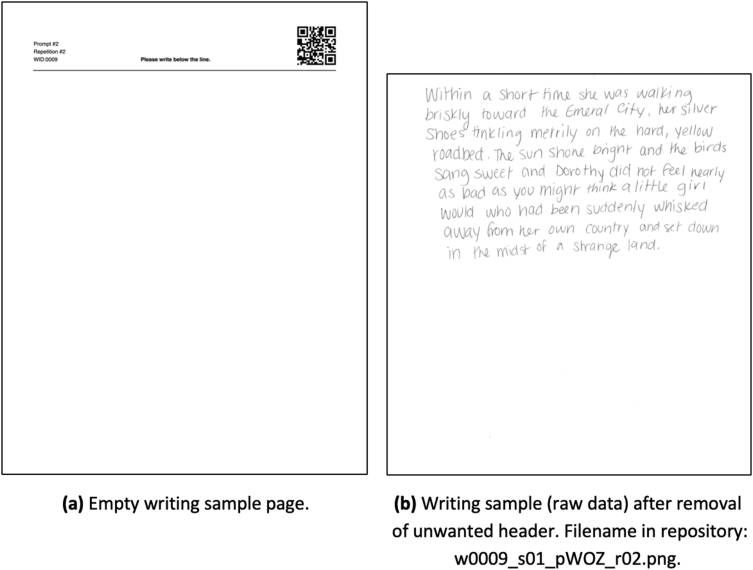
Handwriting collection pages shown before and after data collection and cropping. Pictured is the page for Writer Identification Number (WID) 0009 during their first session where they wrote their second repetition of the *Wizard of Oz* prompt. Details regarding shorthand and naming structure are included in Section 2.

Participants provided writing samples on letter-sized paper that was blank except for a header, depicted in Fig. 1a. The header includes a writer identification number, the prompt to be written, and repetition number of the prompt within the session. All of this information, along with the session number, is embedded in a QR code printed in the upper right corner of the page.

Sample pages were scanned and file names for the images were generated from the text strings stored in the QR codes. The raw data to be captured is the handwriting sample located below the header. Using ImageMagick [1], data were separated from the header by rotating each image clockwise by 0.5° (to offset systematic scan rotation), identifying the printed black line, replacing it the with white pixels, and cropping the top 450 pixels off of each scanned sample image. The raw data images, as in Fig. 1b, are available for use and are stored in the public repository. In total, there are 2430 images available, produced by 90 participants.

In addition to the writing sample images, demographic and session specific information is included in tables in the repository. These data were collected from participants through a series of surveys, one completed at each data collection session. Demographic information, such as gender, dominant hand, age group, and location of third grade education, was collected on the first survey. A set of session specific questions, capturing the current time, date, and location, were included in all three surveys. The second and third surveys include the session specific questions only, and are identical aside from their titles. The surveys given at the first and second sessions are pictured in Figure A.1 in the Appendix.

The first session survey, in Appendix Figure A.1a, asks participants to provide demographic information, some of which is not included in the data tables in the repository. Variables such as initials, ethnicity, location of data collection, and highest level of education are omitted. Initials were collected only for internal quality control. The others are not identifiable on their own, but researchers had concerns regarding the confidentiality of study participants given all of the demographic and session specific information considered together.

Our participant group includes 53 individuals who identify as female and 37 as male. Out of the 90 total participants, 79 are right-handed, 10 are left-handed, and one identified as ambidextrous. Over half of the participants' third grade education took place in the Midwestern United States.

**Fig. 2:**
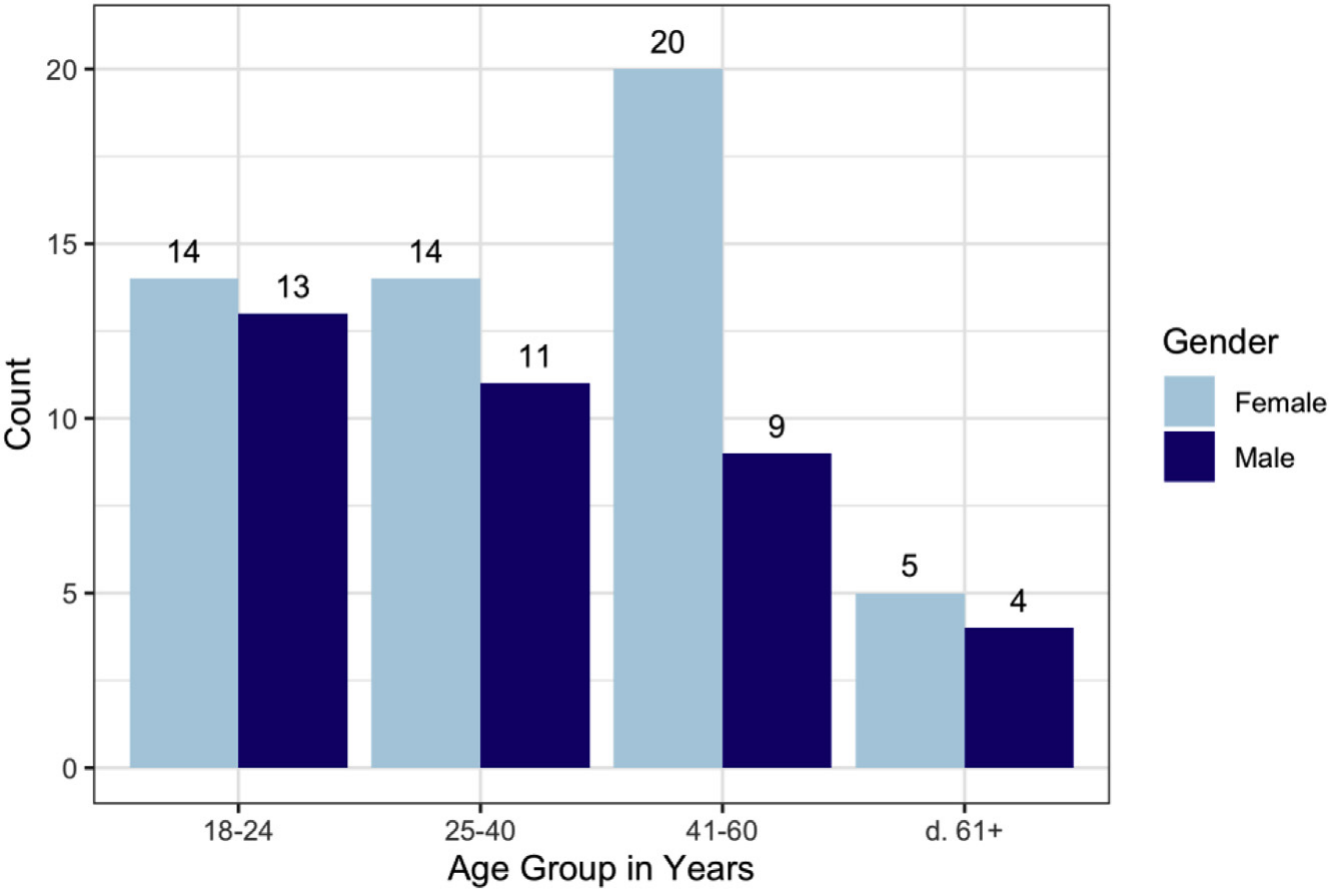
Number of participants in each age group colored by gender identity.

Participant ages are relatively equally spaced across the age categories, shown in Fig. 2 with gender breakdown. Most of the male participants are from younger age groups.

**Fig. 3:**
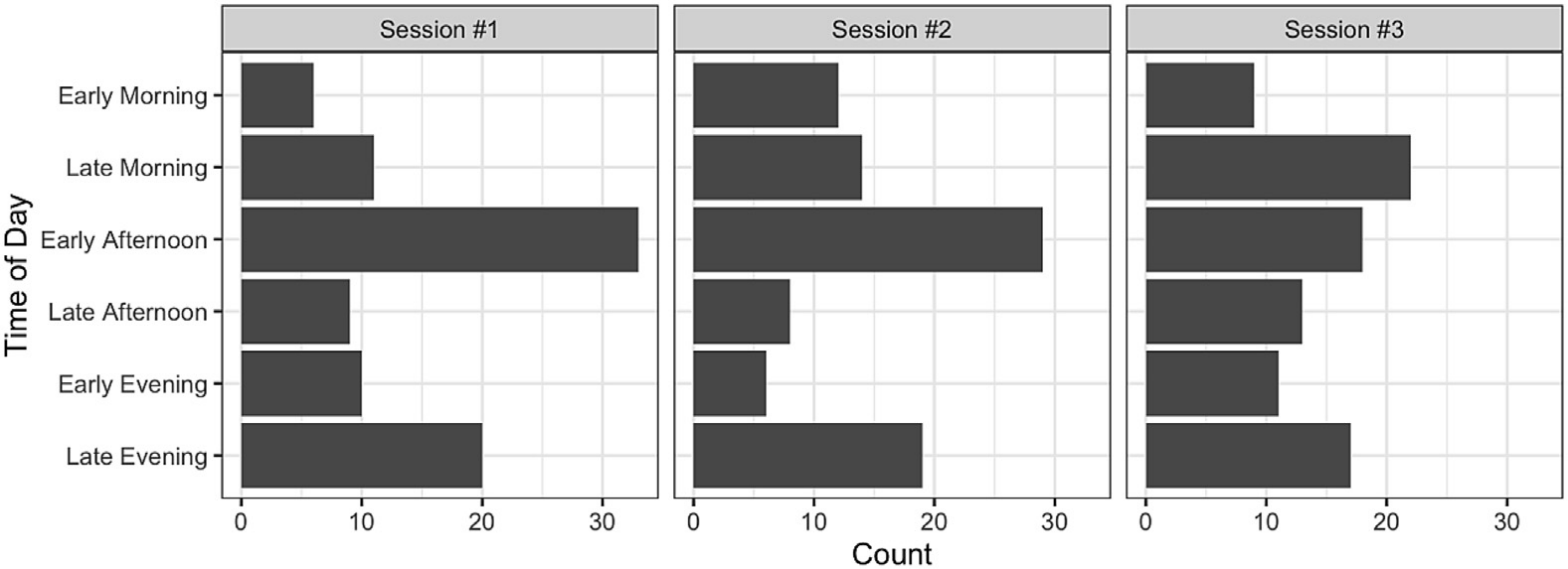
The number of participants who completed their data collection sessions during each time category. One participant left the time field incomplete on the first session survey, and two on the second session survey. These three participants are omitted from the figure.

Session specific information for each data collection session was recorded. The date of each session can be used to investigate the number of days that passed between sessions for each participant. The general time of day that each session was completed was recorded and is summarized below in Fig. 3. The definition of each time group is given in the survey of Figure A.1.

## 2 Experimental design and methods

### 2.1 Experimental design

Recruitment was done primarily via a CSAFE mailing list, in person at Center wide meetings, and by reaching out to personal contacts. The researchers promoted enrolment in cohorts so that materials could be mailed to groups of participants in batches. This method of enrolment lead to location-based participation, and as noted in the previous section, most writers learned to write in the Midwestern United States. The authors acknowledge the bias that can arise from such sampling and encourage users of these data to be mindful of the recruitment technique in their analyses and reporting.

Table 1 gives an overview of the study design skeleton for 180 participants. As writers enrolled in the study, they were assigned a writer identification number (WID) and followed the prompt ordering set forth by the design. This dataset includes samples from 90 unique writers, completing half of the design. The WIDs associated with them are non-consecutive due in part to the sequential WID assignment while cycling through treatments (i.e. 0001, 0061, 0121, 0002, 0062, 0122, etc.).

**Table 1:** A summary of the data collection structure indicating the order in which participants were asked to transcribe prompts at each session. Details regarding shorthand are included below.

	Session #1	Session #2	Session #3
**Treatment A** WID: 0001–0060	1. LND 2. WOZ 3. PHR	1. WOZ 2. PHR 3. LND	1. PHR 2. LND 3. WOZ
**Treatment B** WID: 0061–0120	1. WOZ 2. PHR 3. LND	1. PHR 2. LND 3. WOZ	1. LND 2. WOZ 3. PHR
**Treatment C** WID: 0121–0180	1. PHR 2. LND 3. WOZ	1. LND 2. WOZ 3. PHR	1. WOZ 2. PHR 3. LND

Participants completed three data collection sessions, each at least three weeks apart. As depicted in Table 1, writing prompt orders were cycled such that each treatment group is assigned each of the three prompt orderings over the course of their three data collection sessions. We use the following shorthand to indicate prompt content in the image headers prior to cropping and the file naming structure.

• LND indicates *The London Letter*, a common handwriting exemplar incorporating every letter of the alphabet, each number 1–9, and common punctuation [2].
• WOZ indicates an excerpt from *The Wonderful Wizard of Oz* by L. Frank Baum [3].
• PHR indicates the short common phrase “The early bird may get the worm, but the second mouse gets the cheese.”

At each data collection session, participants were provided an instruction page indicating their prompt order assignment along with the prompt contents. In addition to a survey and three writing prompts, a randomly generated name was given as a signature prompt during each session. Participants were asked to invent a signature for that random name and copy it down (step six in the instructions). The simulated signatures are not part of this data set and are omitted from the procedural descriptions here. A sample instruction page is given in Figure A.2 in the Appendix.

## 2.2 Methods

All writing sample page headers, including QR codes with embedded strings, were systematically generated prior to participant enrolment using R Markdown [4]. Prior to each data collection session, participants received a sample packet (large envelope) including all necessary materials to complete the data collection. Packets were delivered by mail or in-person depending on participant location. Each packet includes an instruction page, a survey, a blue BIC pen, and writing sample pages with headers to indicate prompt content and repetition number. The participant's name appears on an outer shell encompassing the packet. The packet and materials contained within are non-identifiable, marked only with the assigned writer identification number (WID).

Researchers facilitated the first data collection session for every participant, either in-person or via conferencing software, ensuring any questions regarding the informed consent form were addressed. Participants removed and discarded the outer shell including their name, completed the session according to the instruction page (example in Figure A.2), and returned their packets to the researchers.

Physical survey and writing sample pages were removed from the packets, scanned at 300dpi using an Epson DS-6500 document scanner, and stored. A Shiny application [5] was developed in R [6] to facilitate survey data entry and automated image naming. Survey images were loaded in one pane of the application, data were entered into fields of another pane, and the entries were automatically formatted and stored in a data table. The handwriting samples were also loaded in a viewing pane of the application and the ‘pyzbar’ Python library [7] was used to read the QR code printed on the images. A text string embedded in each QR code served as the basis for an automatically assigned file name in the format of wAAAA_sBB_pCCC_rDD.png, where,

• AAAA is a four digit WID (between 0001 and 0180),
• BB is a two digit session number (01, 02, or 03),
• CCC is the three letter prompt shorthand (LND, WOZ, or PHR, see Section 2.1),
• and DD is a number (01, 02, or 03), representing the repetition of a particular prompt in a given session.

Code used to generate the application can be found within the GitHub repository for the Center for Statistics and Applications in Forensic Evidence [8]. After the QR codes were read for naming by the application, the header no longer provided any use and was cropped off of each writing sample as described in Section 1.
