# Supplementary material for: A database of handwriting samples for applications in forensic statistics
Source: Data Brief. 2019 Dec 31;28:105059. doi: 10.1016/j.dib.2019.105059 (PMC6970143; doi:10.1016/j.dib.2019.105059)
Supplement: Multimedia component 2 [file mmc2.docx]

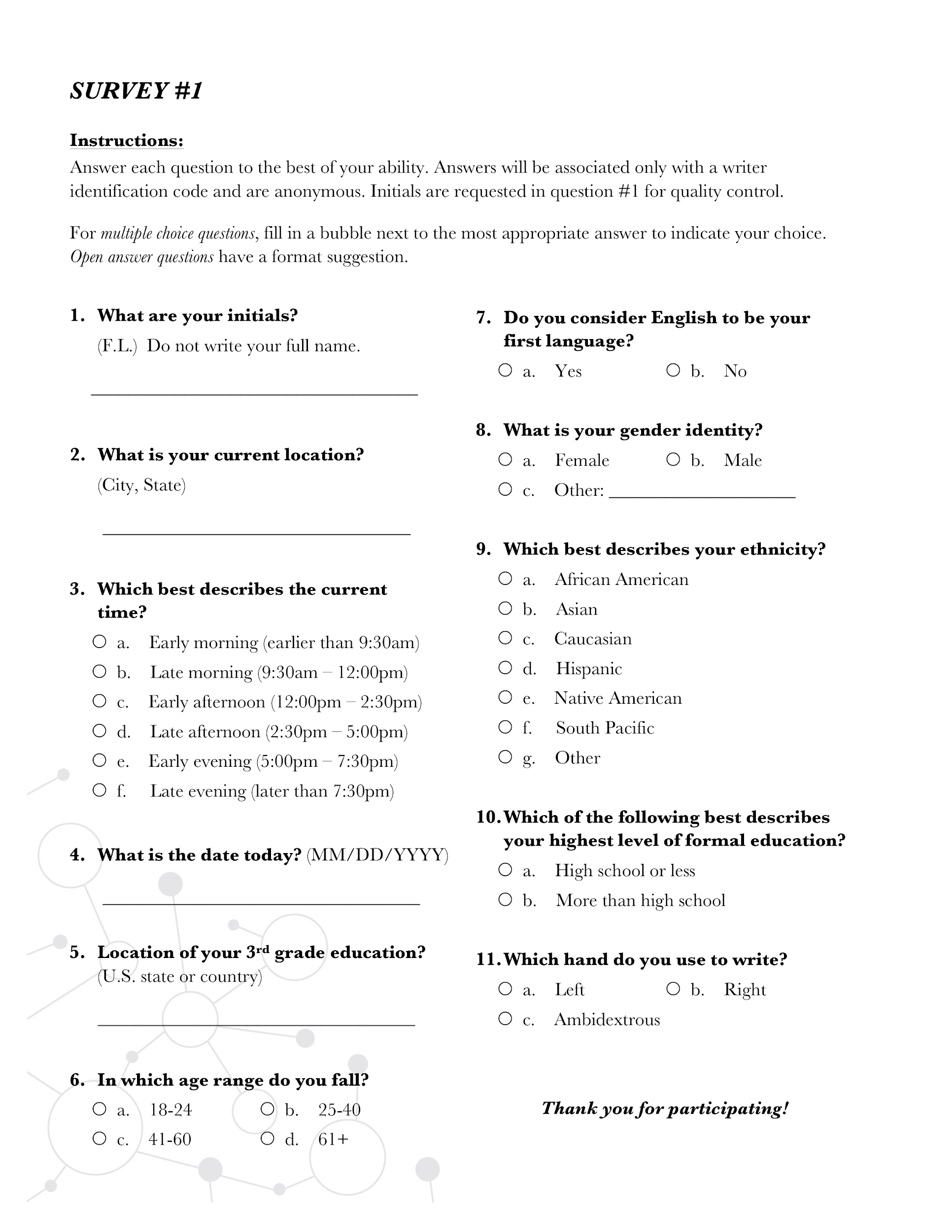
**Appendix**

**Figure A.1 (a).** Survey given to every participant at the first data collection session. Includes demographic and situational information.


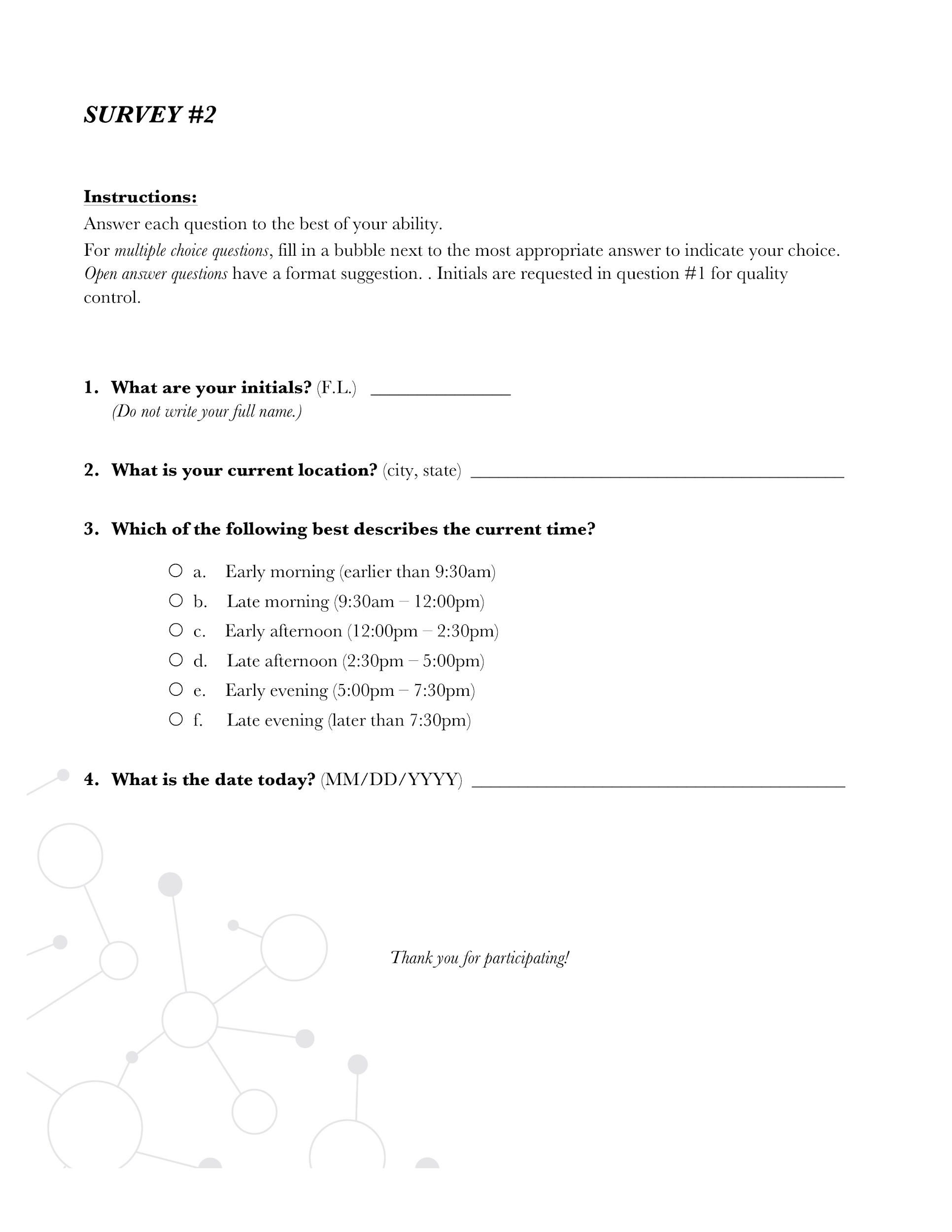


**Figure A.1 (b).** Survey given to every participant at the second data collection session. Includes information about the circumstances under which the second collection happened. Same questions as in Survey #3 given at the third session.

**
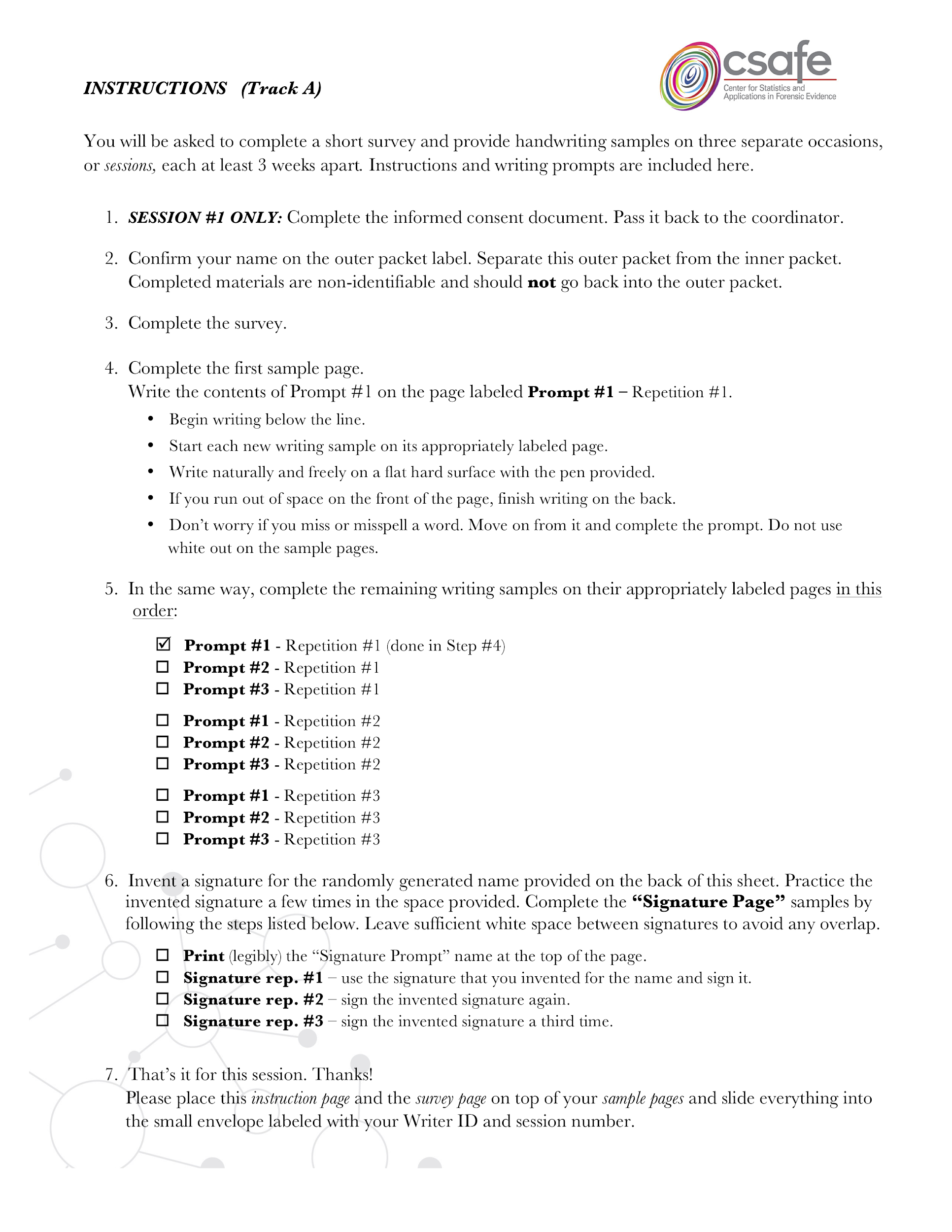
**

**Figure A.2 (a).** Front of a sample instruction page included in every data collection packet.

**
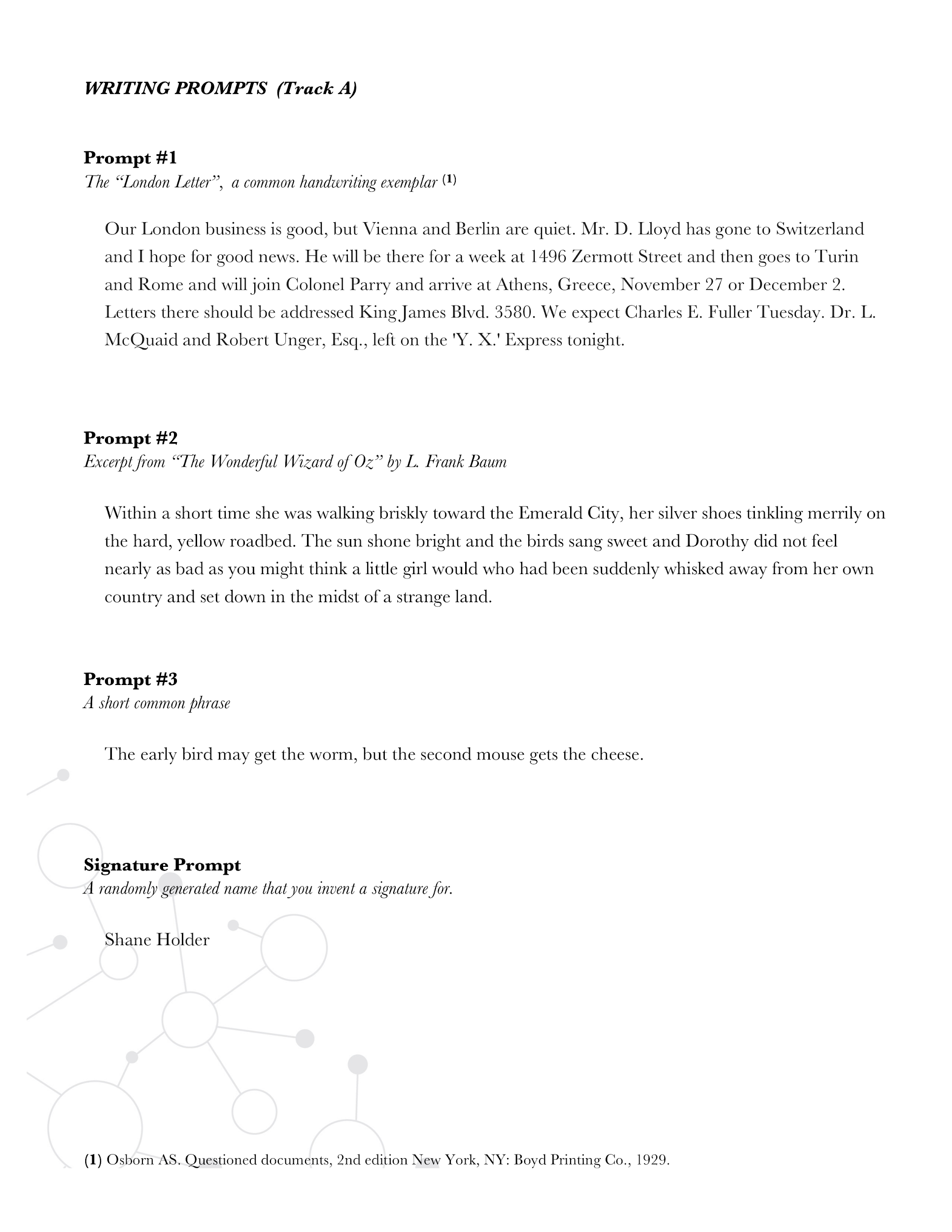
**

**Figure A.2 (b).** Back of a sample instruction page included in every data collection packet.
